# Supplementary figures and images for: Ancient co-option of an amino acid ABC transporter locus in Pseudomonas syringae for host signal-dependent virulence gene regulation
Source: PLoS Pathog. 2020 Jul 16;16(7):e1008680. doi: 10.1371/journal.ppat.1008680 (PMC7386598; doi:10.1371/journal.ppat.1008680)

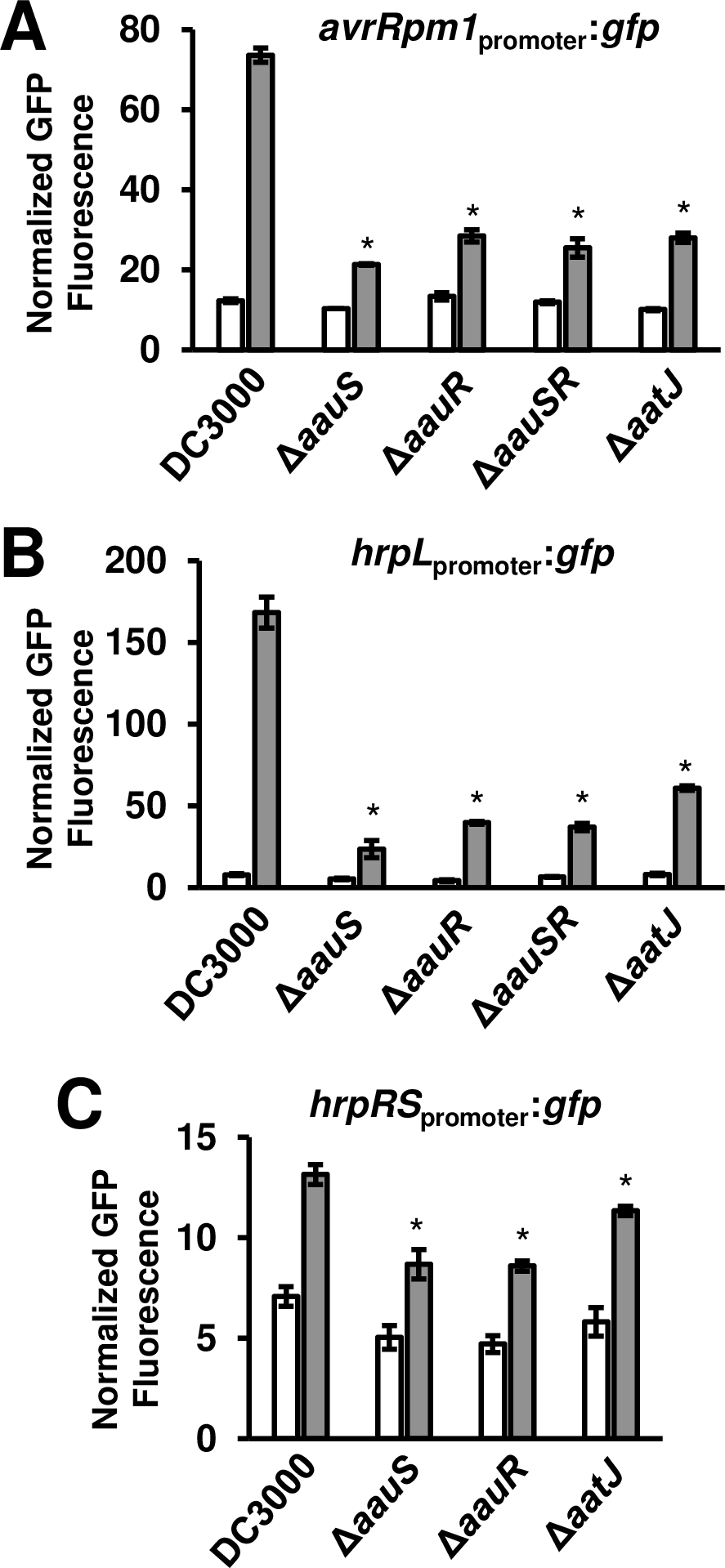

Supplement: S1 Fig — GFP fluorescence of DC3000, ΔaatJ, ΔaauS, ΔaauR and ΔaauSΔaauR strains carrying (A) avrRpm1promoter:gfp, (B) hrpLpromoter:gfp or (C) hrpRSpromoter:gfp reporter plasmids. Bacteria were cultured in a minimal medium (MM) with 10 mM fructose (open bars) and 10 mM fructose plus 200 μM glutamate (filled bars) for 6 hours. Graphed are means ± SE of GFP fluorescence normalized to OD600 and background fluorescence from empty vector strains; n = 3. Asterisks denote significant difference based on two-sample t-test comparison with DC3000 treated with fructose and aspartate, P < 0.05. Data are representative of three independent experiments. (TIF) [file ppat.1008680.s001.tif]

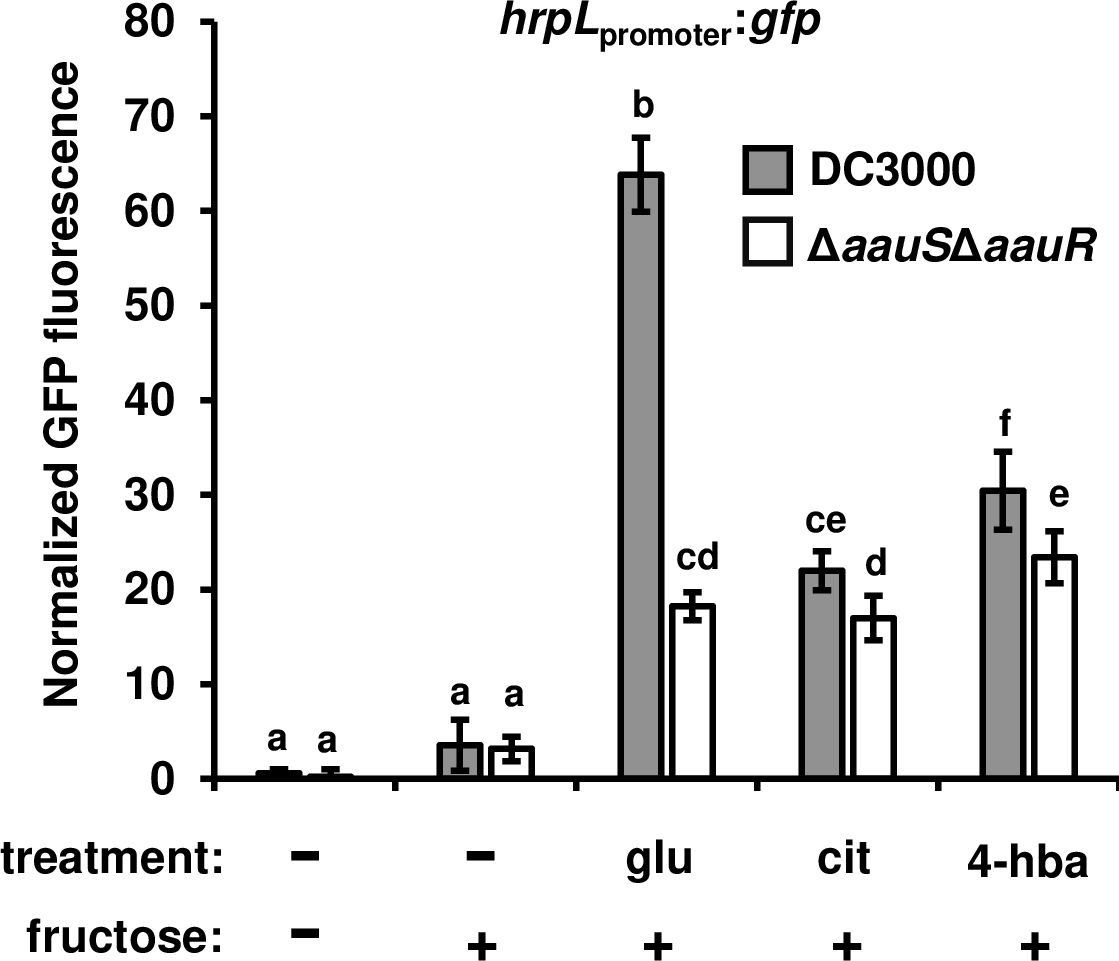

Supplement: S2 Fig — GFP fluorescence of DC3000 and ΔaauSΔaauR carrying hrpLpromoter:gfp reporter plasmids. Bacteria were cultured for eight hours in minimal medium containing 10 mM fructose and with or without 200 μM glutamate, citrate, or 4-hydroxybenzoic acid. Graphed are means ± SD of the change in GFP fluorescence at T = 8 hours post-treatment. Fluorescence values were normalized to OD600 and background fluorescence from empty vector strains; n = 12. Data are pooled from 3 independent experiments, n = 4 per experiment. Small-case letters denote statistical significance based on ANOVA with Tukey’s post-hoc HSD test, P < 0.05. (TIF) [file ppat.1008680.s002.tif]

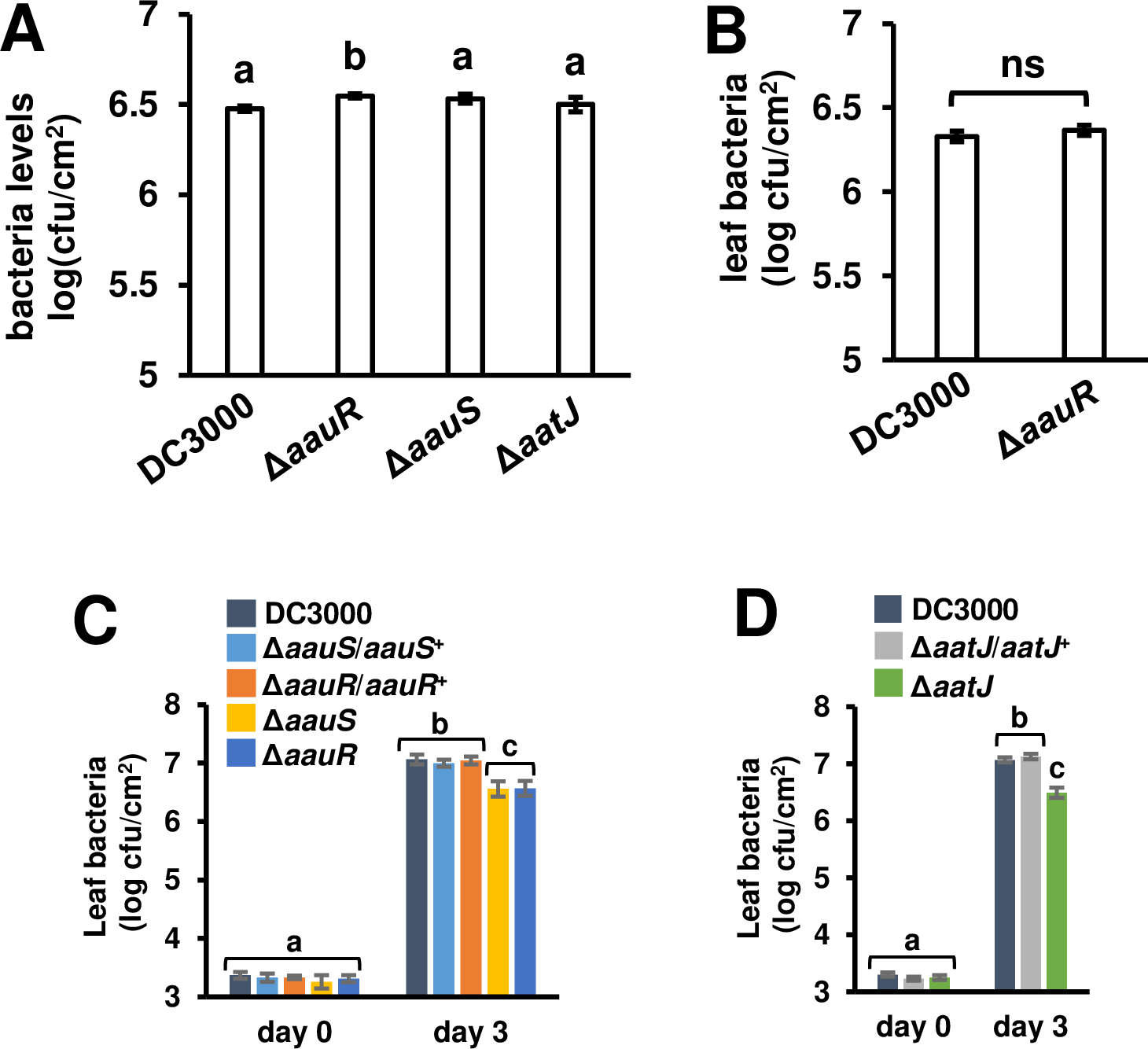

Supplement: S3 Fig — (A) 1 x 10^8^ cfu/mL of DC3000, ΔaauR, ΔaauS or ΔaatJ were syringe-infiltrated in Arabidopsis leaves. Leaf bacteria populations were measured by serial dilution plating of leaf extracts six hours post-infection. Graphed are means ± SE of bacteria colonies isolated from infected tissue, n = 3. Abbreviation ns is not significant based on ANOVA with Tukey’s HSD, α = .05. (B) 5 x 10^8^ cfu/mL of DC3000 or ΔaauR were syringe-infiltrated into Arabidopsis leaves. Leaf bacteria populations were measured by serial dilution plating of leaf extracts 6 hours post-infection. Graphed are means ± SE of bacteria colonies isolated from infected tissue, n = 3. Abbreviation ns is not significant based on t-test, P = .444. (C-D) 1 x 10^6^ cfu/mL of DC3000, ΔaauS, ΔaauR, or ΔaatJ and respective complemented strains were syringe-infiltrated into Arabidopsis leaves. Leaf bacteria populations were enumerated on day 0 and day 3 by serial dilution plating of leaf extracts. Graphed are log-transformed means ± SE of bacteria colony-forming units (cfus) isolated from infected tissue, n = 6 for day 0 and n = 8 for day 3. Small-case letters denote significance groupings based on ANOVA with Tukey’s HSD, P < 0.01. Data shown were pooled from two independent experiments. (TIF) [file ppat.1008680.s003.tif]

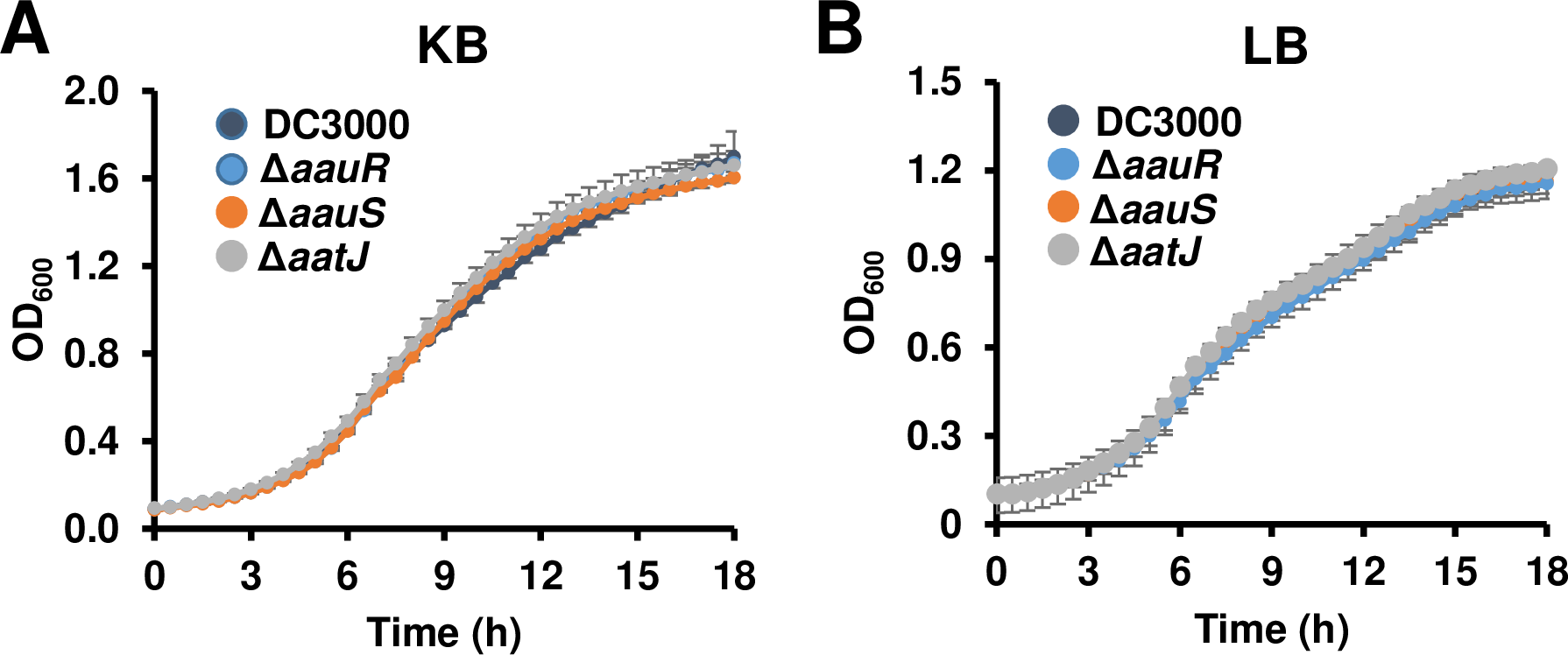

Supplement: S4 Fig — DC3000, ΔaatJ, ΔaauS, and ΔaauR were individually inoculated into 100 μL of (A) Lysogeny broth (LB) or (B) King’s B (KB) broth and grown for 24 hours at 28°C. OD600 readings of each well were taken at 30 min intervals. Graphed are means ± SD of OD600 readings, n = 3. Results are representative of 3 independent experiments. (TIF) [file ppat.1008680.s004.tif]

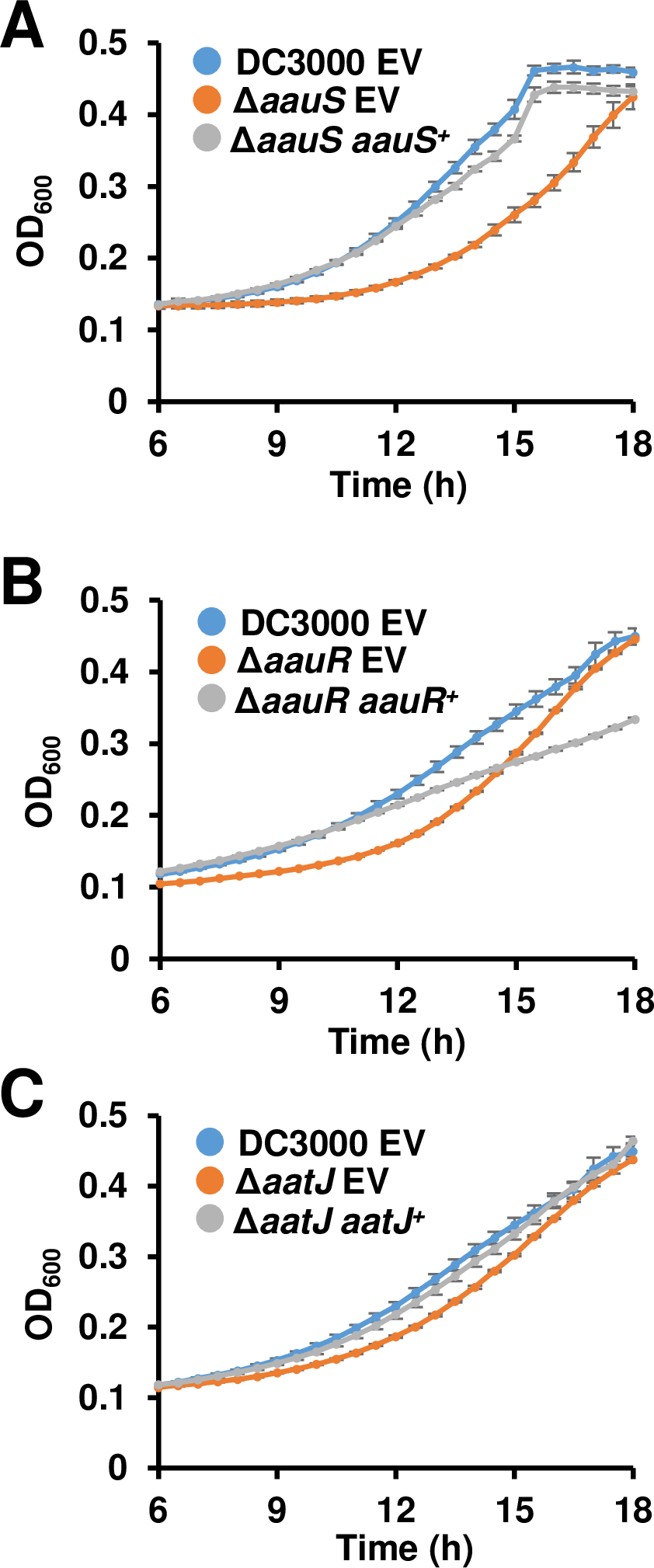

Supplement: S5 Fig — (A) DC3000 and ΔaauS carrying empty vector (EV) pME6010 or aauS::pME6010; (B) DC3000 and ΔaauR carrying empty vector (EV) pME6010 or aauR::pME6010; and (C) DC3000 and ΔaatJ carrying empty vector (EV) pME6010 or aatJ::pME6010 were inoculated into 100 μL of M9 medium supplemented with 10 mM glutamate as the sole carbon source. Optical density of each culture was measured every 30 minutes from 6 to 18 hours post-inoculation. Graphed are means ± SD of OD600 readings, n = 4. Results are representative of 3 independent experiments. (TIF) [file ppat.1008680.s005.tif]

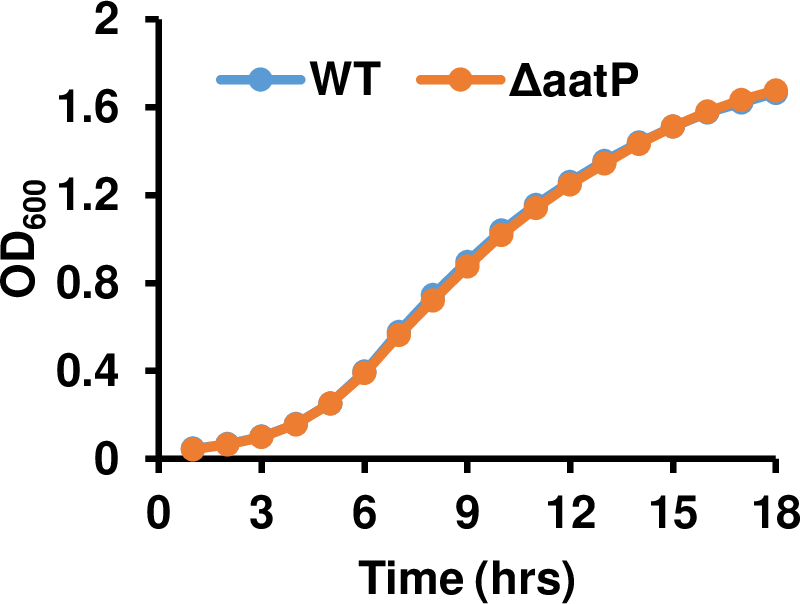

Supplement: S6 Fig — Timecourse analysis of DC3000 and ΔaatP growth in King’s B (KB) broth. Cultures were inoculated at an optical density at λ = 600 nm (OD600) of 0.05 and grown for 24 hours at 28°C. Graphed are means ± SD of OD600 readings, n = 3. Results are representative of 3 independent experiments. (TIF) [file ppat.1008680.s006.tif]

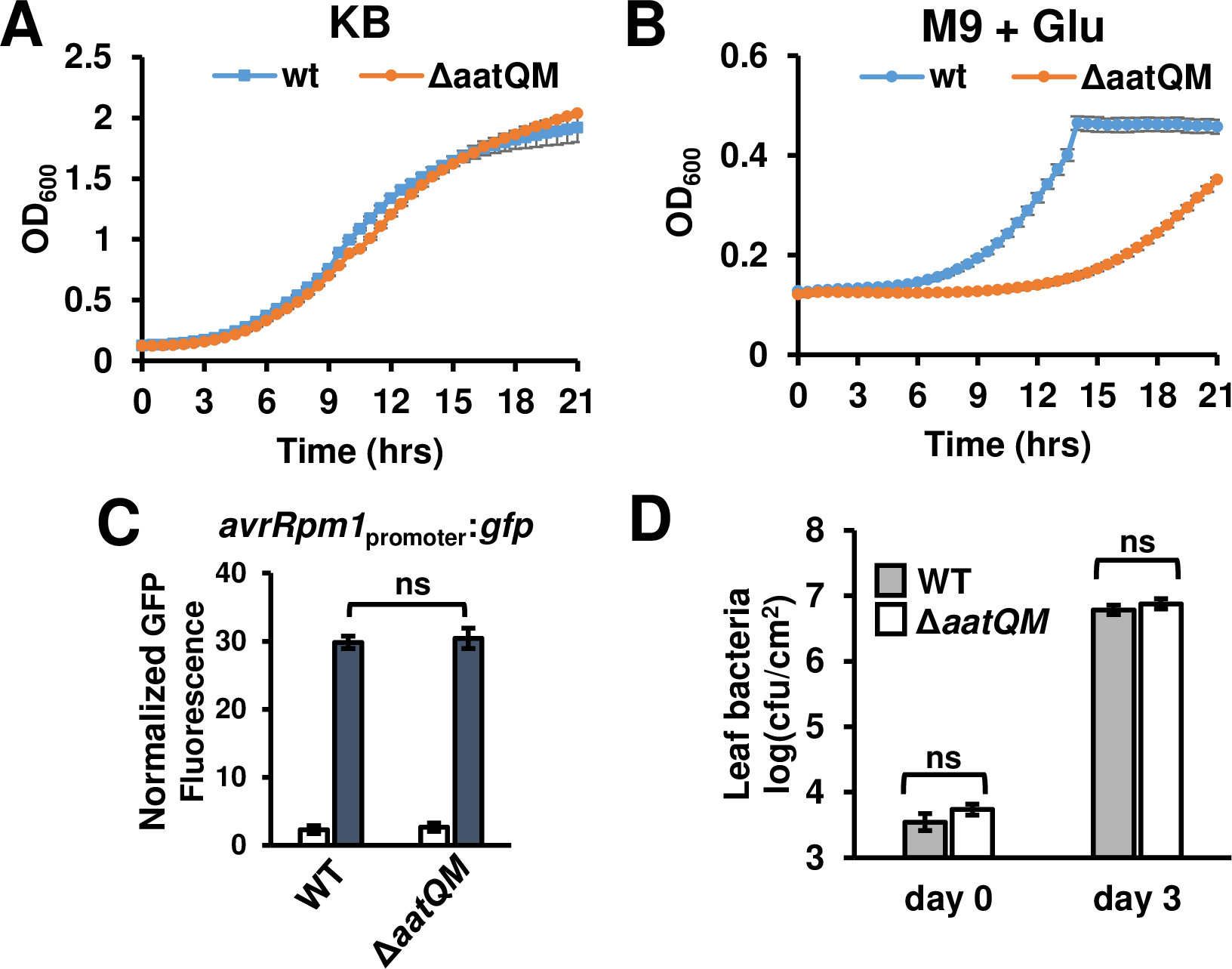

Supplement: S7 Fig — Growth of DC3000 and ΔaatQaatM strains in KB medium (A) or M9 minimal medium with glutamate as a sole carbon and nitrogen source (B). Cultures were inoculated at an optical density (λ = 600 nm) of 0.05 and grown for 24 hours at 28°C in wells of a 96-well plate. Optical density at λ = 600 nm (OD600) readings of each well were taken at 30 min intervals. Graphed are means ± SD of OD600 readings, n = 3. (C) Analysis of GFP fluorescence from DC3000 and ΔaatQaatM avrRpm1promoter:gfp reporter strains incubated with 10 mM fructose (open bars) or 10 mM fructose and 200 μM glutamate (filled bars) for 6 hours. Graphed are means ± SD of GFP fluorescence normalized to OD600 and background fluorescence from empty vector strains; n = 4. Abbreviation ns is not significant based on t-test with α = 0.05. (D) Growth of DC3000 and ΔaatQaatM strains in Arabidopsis. A 1 x 10^6^ cfu/mL inoculum of each strain was syringe-infiltrated into Arabidopsis leaves. Leaf bacteria populations were enumerated on day 0 and day 3 by serial dilution plating of leaf tissue extracts. Graphed are means ± SE of bacteria from 4 infected plants, n = 3 for day 0 and n = 4 for day 3. Abbreviation ns is not significant based on t-test with α = 0.05. (TIF) [file ppat.1008680.s007.tif]

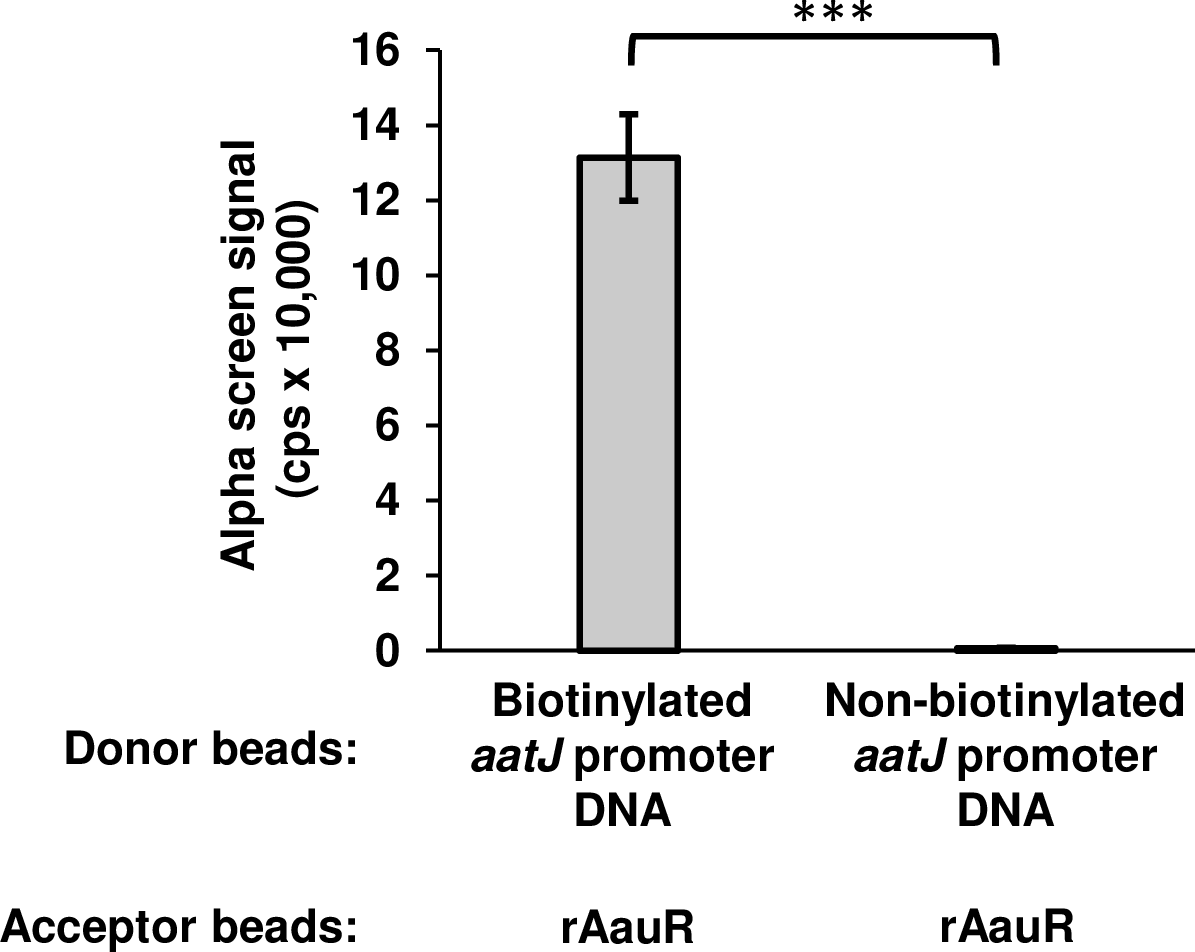

Supplement: S8 Fig — AlphaScreen assay of E. coli-expressed recombinant DC3000 AauR protein binding to 50 bp of aatJ promoter DNA including the Rbm sequence. Assays containing non-biotinylated DNA were included as negative controls to demonstrate specificity of detected interactions. Graphed are means ± SD of luminescence from assay wells, n = 3. Asterisks is P < 0.001 based on t-test. Data are representative of 3 independent experiments. (TIF) [file ppat.1008680.s008.tif]

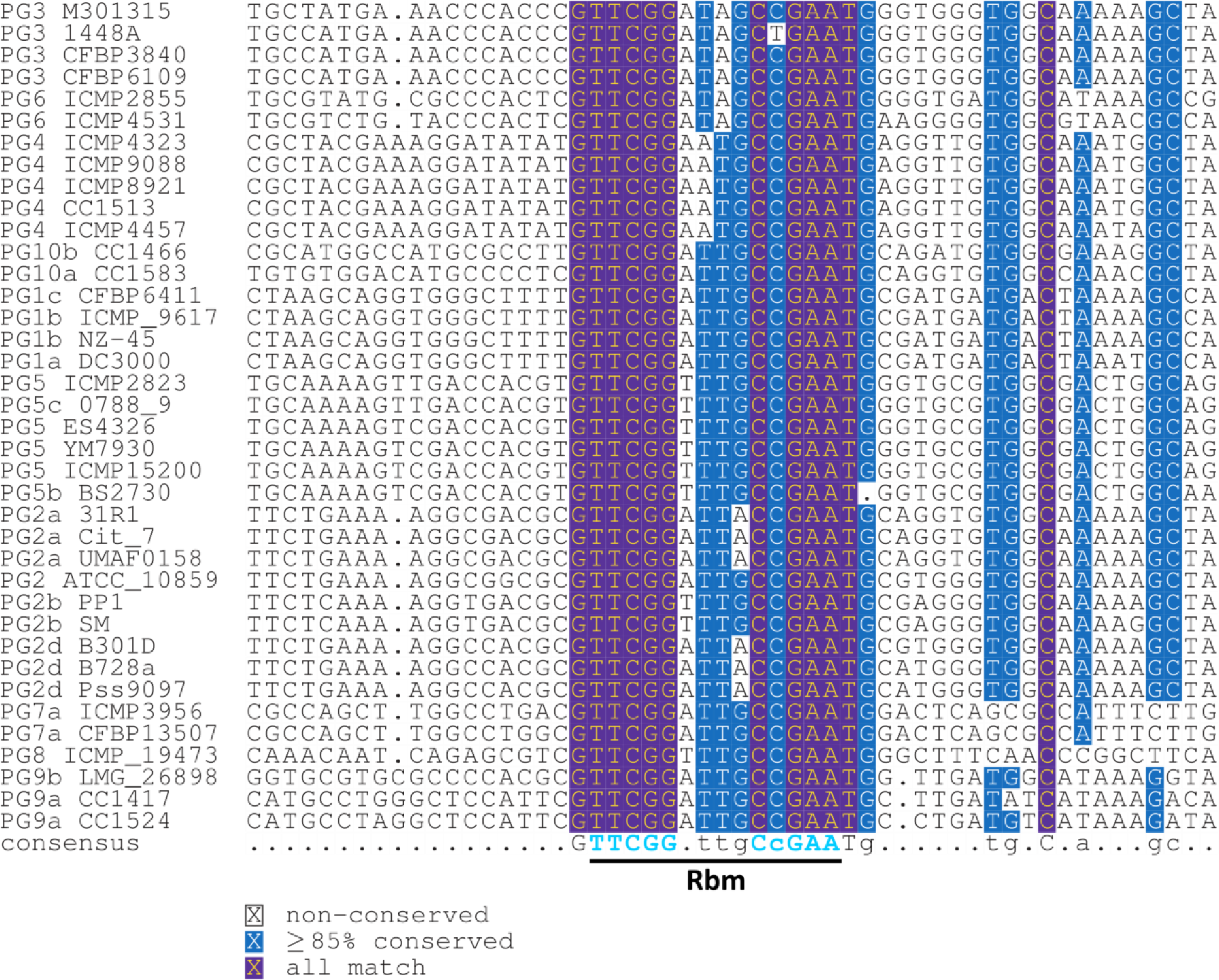

Supplement: S9 Fig — Alignment of genomic DNA containing Rbm upstream of hrpRS from 38 P. syringae isolates. Sequences are labeled with phylogroup and strain name. Full names can be found in S3 Table. Upper and lower case letters below alignment are consensus sequence of nucleotides based on 100% and ≥85% identity, respectively; dots on the consensus line indicate no consensus based on either criteria. Highlighted in blue is AauR-binding motif (Rbm). (TIF) [file ppat.1008680.s009.tif]

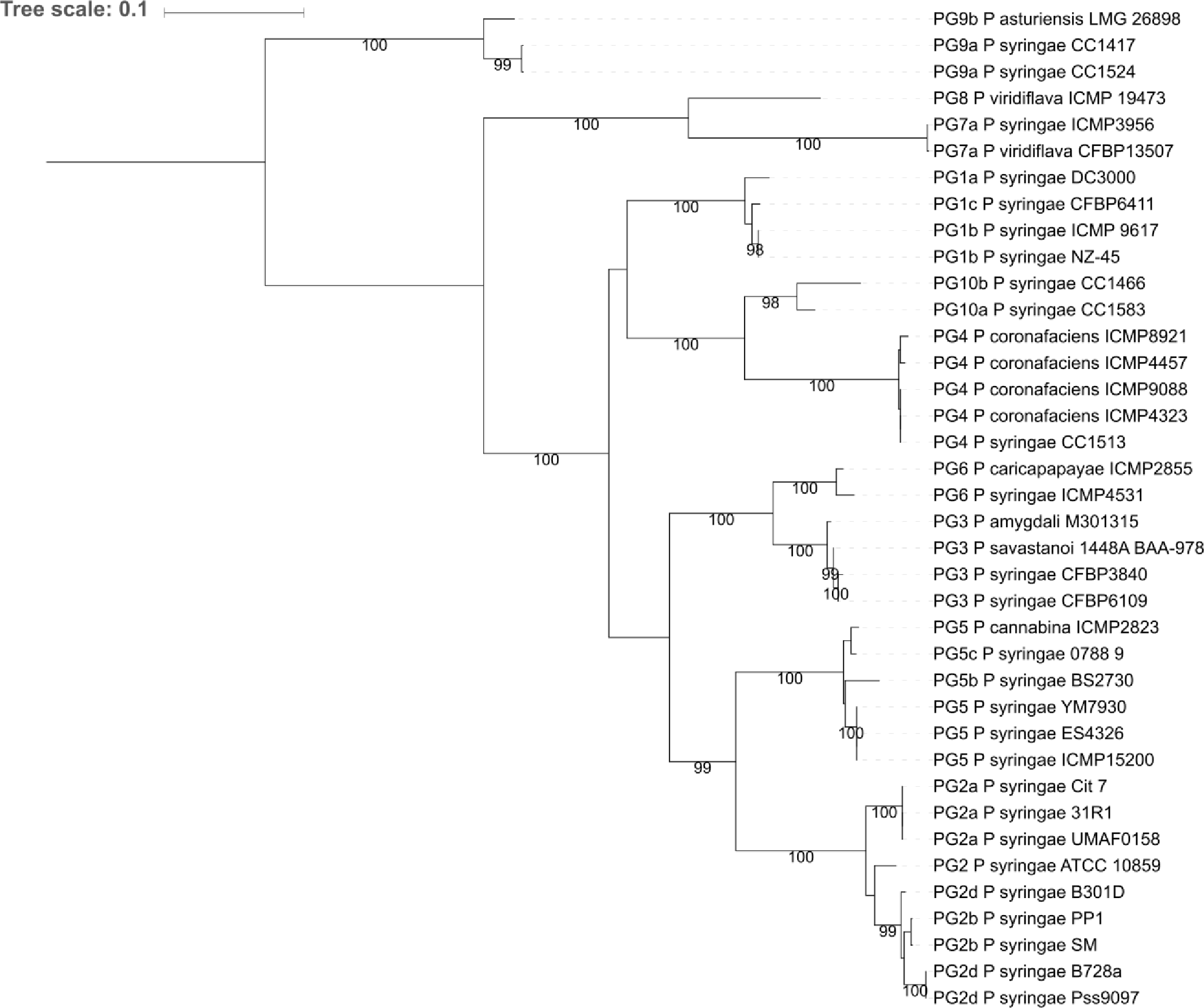

Supplement: S10 Fig — Nucleotide sequences of hrpR from all canonical tripartite type III secretion system encoding P. syringae strains examined in this study are included in the phylogeny. Ultrafast bootstraps (>95% support) calculated by IQ-TREE are reported. Within phylogroup supports are all above the 95% bootstrap support threshold. Branch lengths indicate the number of mutations per site. The tree is artificially rooted on representatives from PG9 for reference. (TIF) [file ppat.1008680.s010.tif]

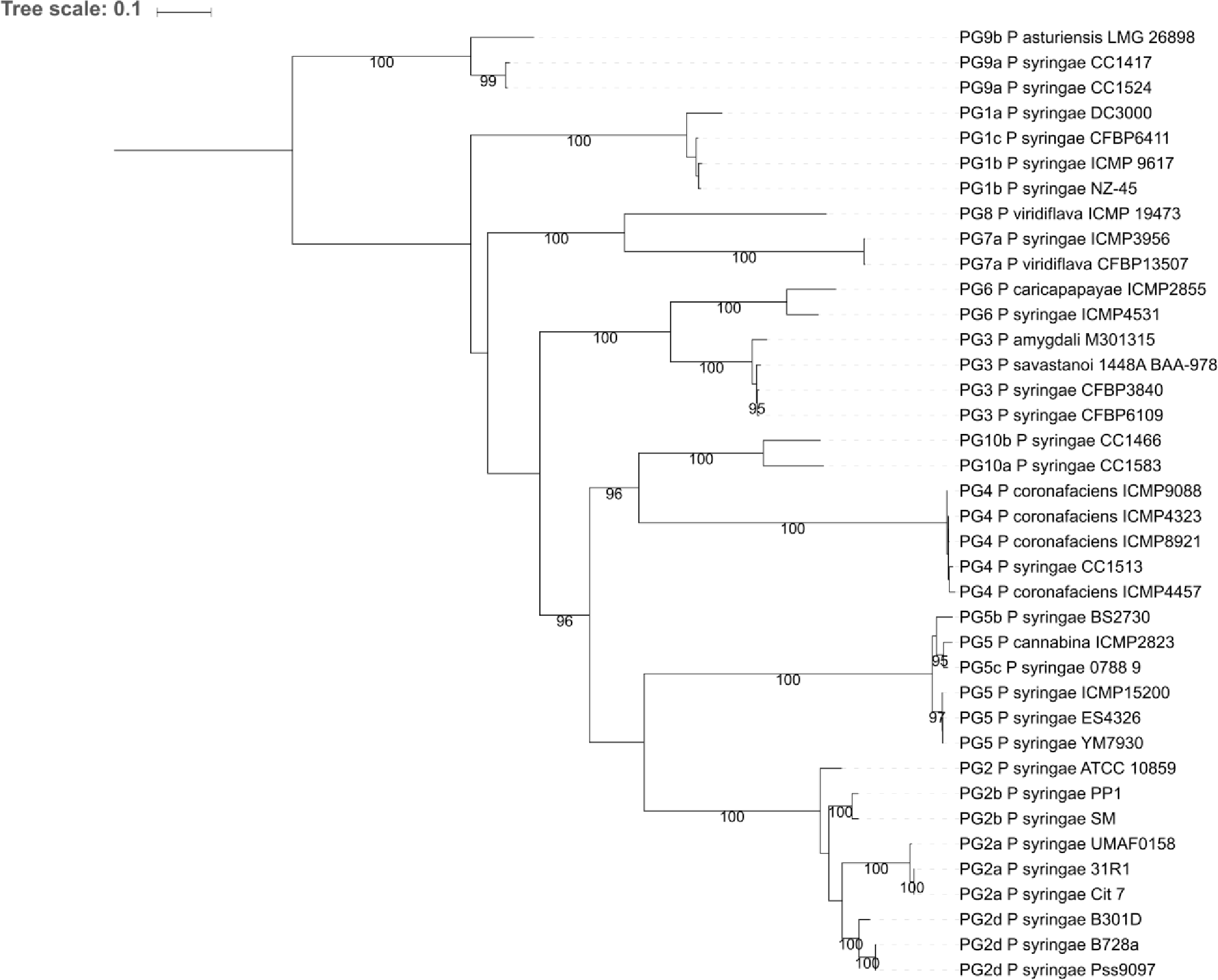

Supplement: S11 Fig — Nucleotide sequences corresponding to the intergenic regions between hrpH and hrpR from all canonical tripartite type III secretion system encoding P. syringae strains examined in this study are included in the phylogeny. Ultrafast bootstraps (>95% support) calculated by IQ-TREE are reported. Within phylogroup supports are all above the 95% bootstrap support threshold. Branch lengths indicate the number of mutations per site. The tree is artificially rooted on representatives from PG9 for reference. (TIF) [file ppat.1008680.s011.tif]

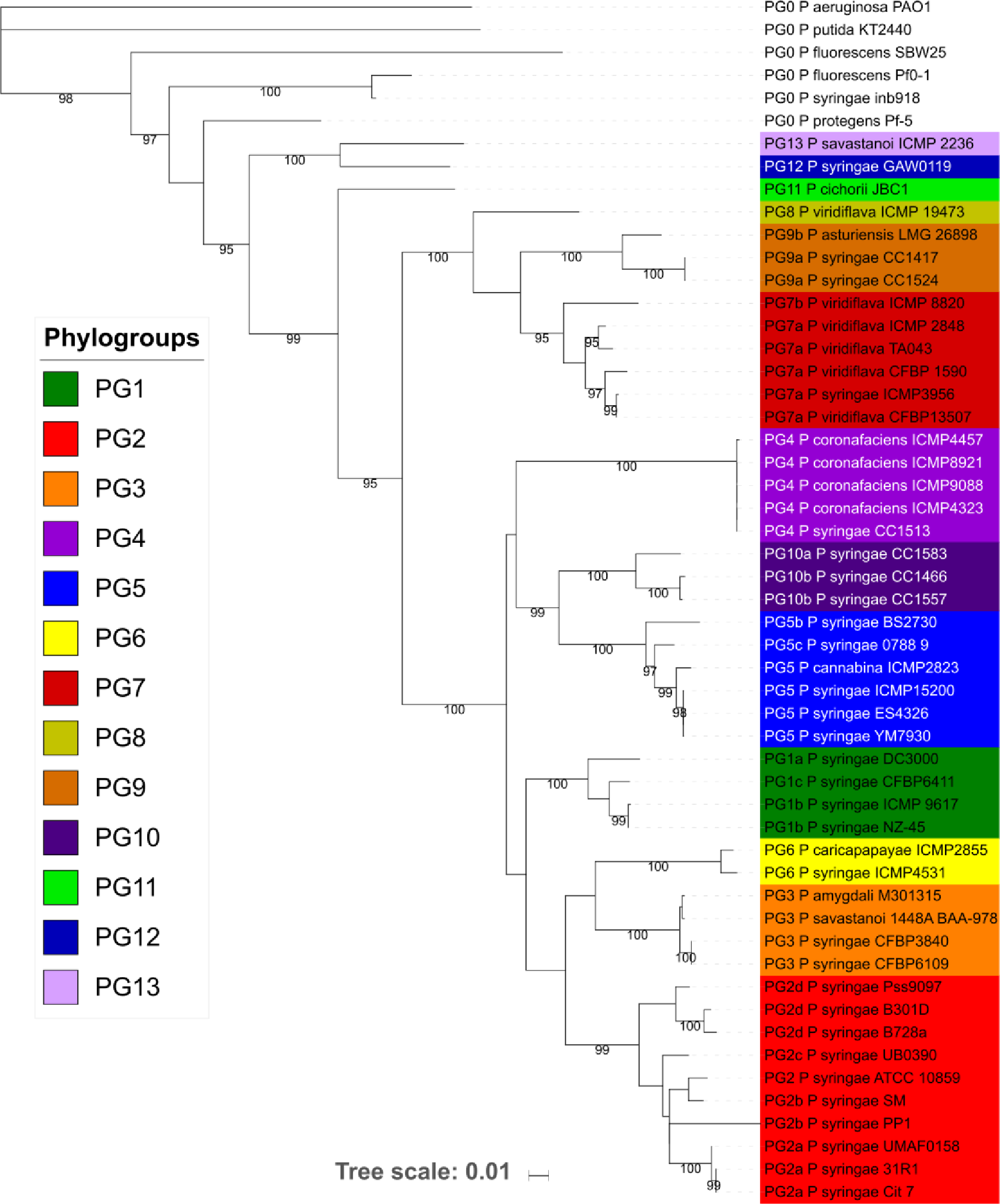

Supplement: S12 Fig — Nucleotide sequences of aatJ from diverse P. syringae isolates and other pseudomonads are included in the phylogeny. Ultrafast bootstraps (>95% support) calculated by IQ-TREE are reported. Within phylogroup supports are all above the 95% bootstrap support threshold. Branch lengths indicate the number of mutations per site. The tree is artificially rooted on branches with representative Pseudomonas spp. outside of P. syringae for reference. (TIF) [file ppat.1008680.s012.tif]

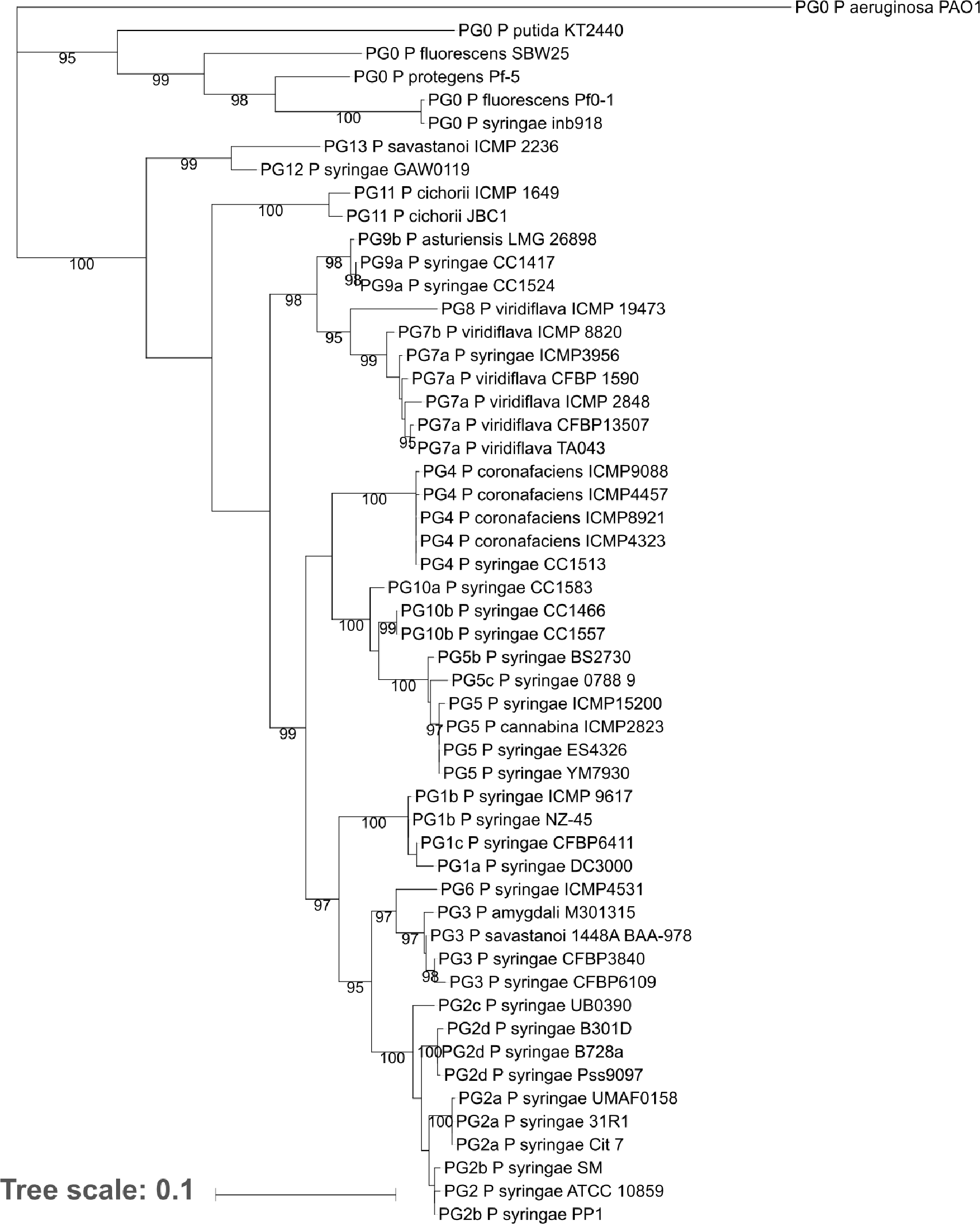

Supplement: S13 Fig — Amino acid sequences of AauS from diverse P. syringae isolates and other pseudomonads are included in the phylogeny. Ultrafast bootstraps (>95% support) calculated by IQ-TREE are reported. Within phylogroup supports are all above the 95% bootstrap support threshold. Branch lengths indicate the number of mutations per site. The tree is artificially rooted on branches with representative Pseudomonas spp. outside of P. syringae for reference. (TIF) [file ppat.1008680.s013.tif]

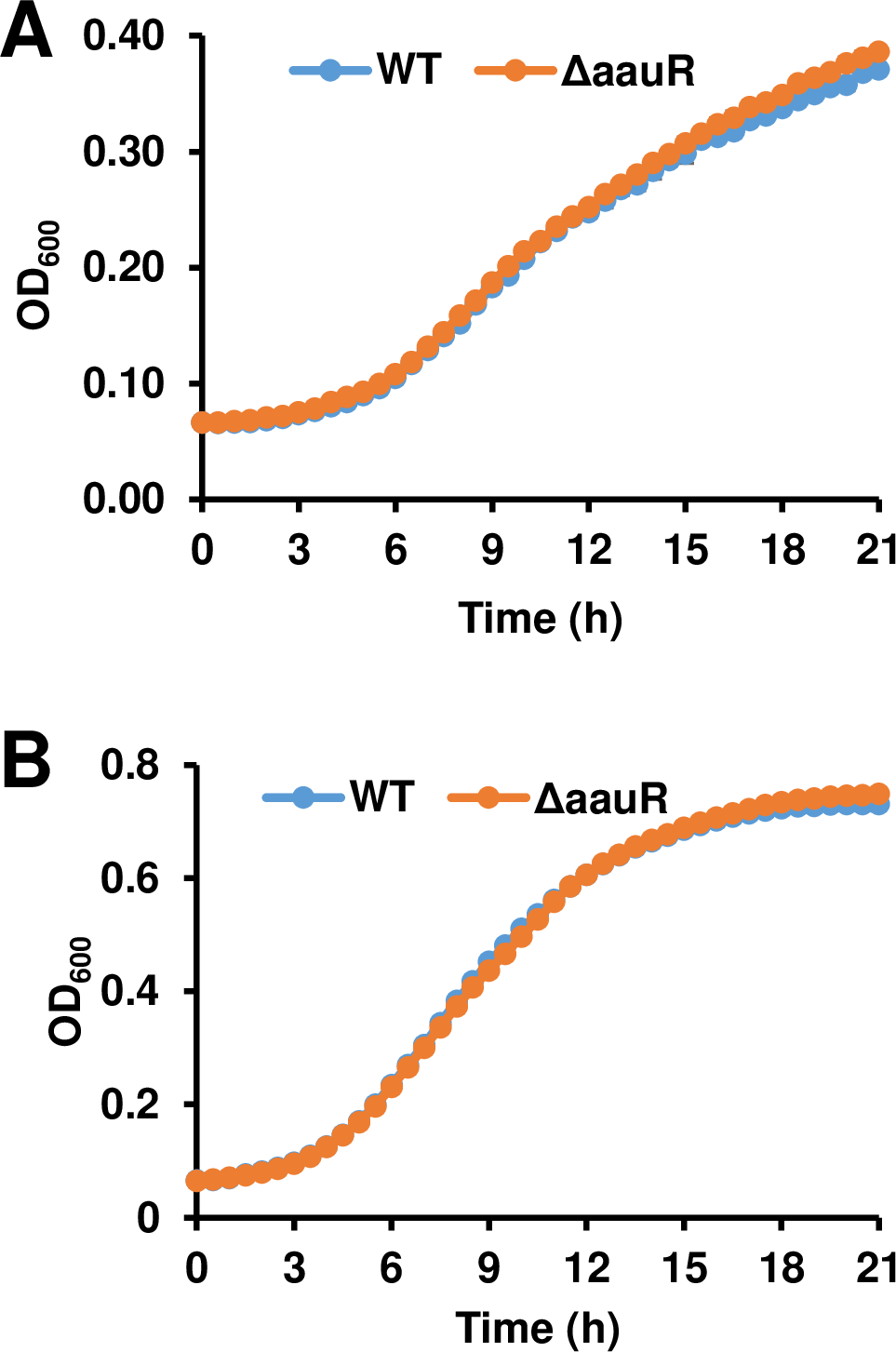

Supplement: S14 Fig — Timecourse analysis of B728a and ΔaauR growth in (A) standard M9 medium supplemented with glucose and ammonium chloride or (B) lysogeny broth (LB). Cultures were inoculated at an optical density at λ = 600 nm (OD600) of 0.05 and grown for 24 hours at 28°C in a Tecan Spark 10M plate reader. Graphed are means ± SD of OD600 readings, n = 3. Results are representative of 3 independent experiments. (TIF) [file ppat.1008680.s014.tif]

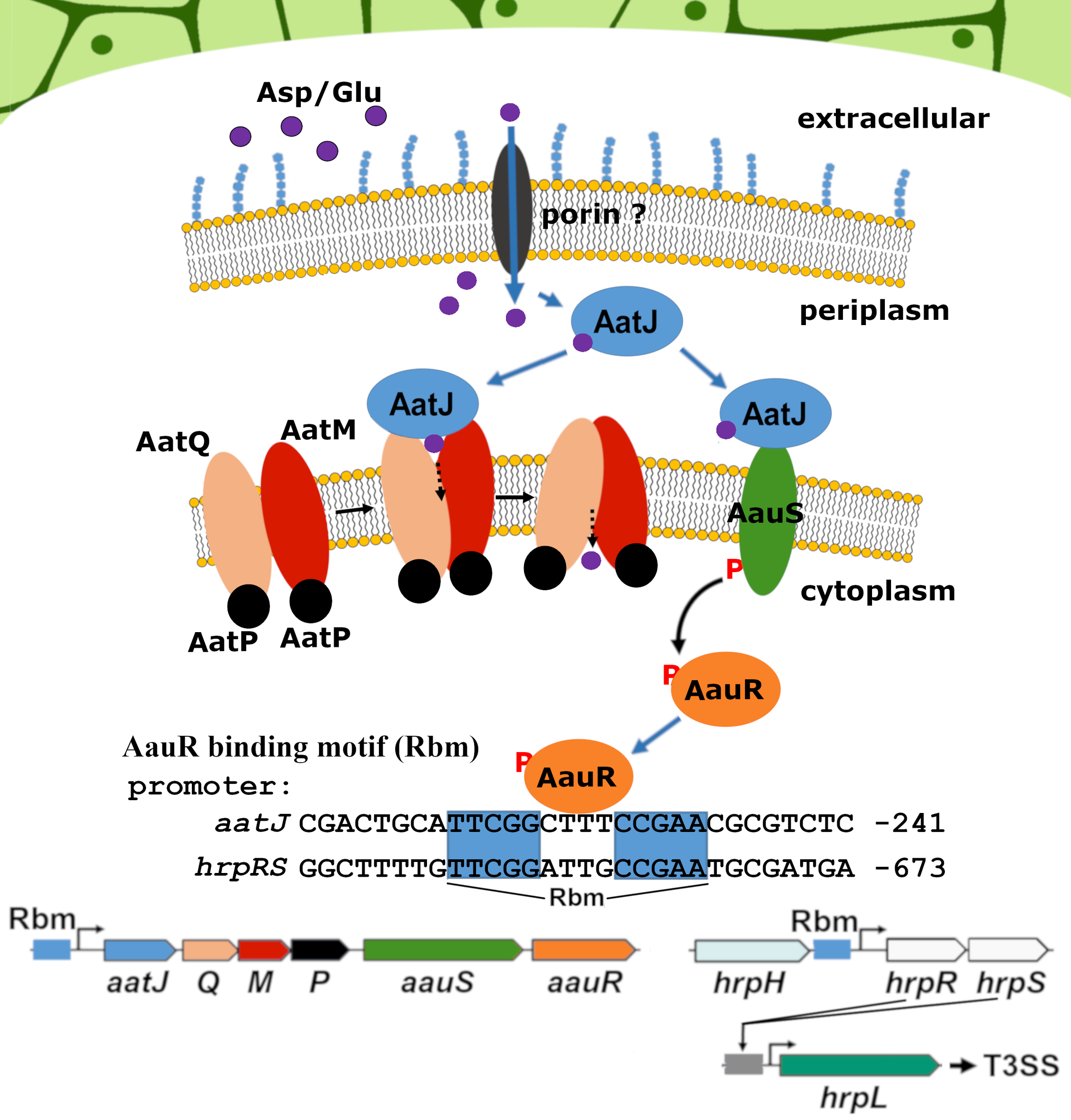

Supplement: S15 Fig — Plant-exuded aspartate and glutamate enter the periplasm, mostly likely through an outer membrane porin, and bind to AatJ, an ABC transporter-associated substrate-binding protein. AatJ, in complex with aspartate or glutamate, binds to AatQM to deliver these metabolites for transport across the inner membrane, and also binds to AauS to activate signaling of the two-component system AauSR. Alternatively, aspartate/glutamate may directly bind to and activate AauS, with AatJ functioning indirectly in promoting AauSR activation (not shown in model). Activated AauS phosphorylates and activates AauR, which in turn binds to a conserved motif (AauR binding motif, Rbm) present within the promoter region of aatJ to increase expression of aat/aau genes. Activated AauR also binds to an Rbm upstream of the operon encoding enhancer-binding proteins HrpR and HrpS. Increased abundance of HrpR and HrpS results in increased expression of the gene encoding the alternative sigma factor HrpL, which in turn activates expression of genes encoding type III secretion system (T3SS) structural components and effectors. See discussion for additional details of model. (TIF) [file ppat.1008680.s015.tif]
