# Supplementary material for: Ancient co-option of an amino acid ABC transporter locus in Pseudomonas syringae for host signal-dependent virulence gene regulation
Source: PLoS Pathog. 2020 Jul 16;16(7):e1008680. doi: 10.1371/journal.ppat.1008680 (PMC7386598; doi:10.1371/journal.ppat.1008680)
Supplement: S1 Table — (PDF) [file ppat.1008680.s016.pdf]

S1 Table. Bacterial strains and plasmids used in this study.

**Strains and plasmids**	**Genotype and relevant characteristics#**	**Reference or source**
**Strains**		
P. syringae		
DC3000	Wild-type DC3000. Rif^r	Lab collection
Δ*aauS*	Strain DC3000 containing a 1580bp in-frame deletion of *aauS* in the chromosome	This study
Δ*aauR*	Strain DC3000 containing a 1070bp in-frame deletion of *aauR* in the chromosome	This study
Δ*aauS* Δ*aauR*	Strain DC3000 containing a 3219bp deletion of *aauS and aauR* in the chromosome	This study
Δ*aatJ*	Strain DC3000 containing a 793bp in-frame deletion of *aatJ* in the chromosome	This study
Δ*aatP*	Strain DC3000 containing a 690bp in-frame deletion of *aatP* in the chromosome	This study
Δ*aatQM*	Strain DC3000 containing a 1300bp deletion of *aatQ and aatM* in the chromosome	This study
ΔRbm	Strain DC3000 containing a deletion of the AauR-binding site Rbm within the HrpR promoter region	This study
B728a	Wild-type. Rif^r	Lab collection
B728a_ Δ*aauR*	Strain B728a containing a 1297bp in-frame deletion of *aauR* in the chromosome	This study
E. coli	*recA pro hsdR*$^-$M$^+$ RP4 2-Tc::Mu-Km::*Tn*7 Sm^r Tp^r	
**Plasmids**		
pEX18Km	Gene replacement vector with MCS from pUC18, *sacB*$^+$ Km^r	(Hoang *et al.* 1998)
pRK600	Helper plasmid used in tri-parental matting. Cm^r	
p18Km-ΔaauS	pEX18Km with a deletion of *aauS* (PSPTO_4175)	This study
p18Km-ΔaauR	pEX18Km with a deletion of *aauR* (PSPTO_4176)	This study
p18Km-ΔaauSΔaauR	pEX18Km with a deletion of both *aauS* (PSPTO_4175) and *aauR* (PSPTO_4176)	This study
p18Km-ΔaatJ	pEX18Km with a deletion of *aatJ* (PSPTO_4171)	This study
p18Km-ΔaatP	pEX18Km with a deletion of *aatP* (PSPTO_4174)	This study
p18Km-ΔaatQM	pEX18Km with a deletion of both *aatQ* (PSPTO_4172) and *aatM* (PSPTO_4173)	This study

p18Km-ΔRbm	pEX18Km with a deletion of AauR-binding site Rbm in the promoter region of *hrpR*	This study
p18Km-ΔaauR_B728a	pEX18Km with a deletion of *aauR* (Psyr_3913)	This study
pPROBE-NT	pBBR1, containing promoterless *gfp*, Km^r	(Miller *et al.* 2000)
$phrpRS_{promoter}$-gfp	pPROBE-NT reporter construct containing the intergenic region between *hrpR* and *hrpH* including the promoter of *hrpRS* fused with a promoterless *gfp*	This study
$phrpR_{promoter}$(ΔRbm)-gfp	pPROBE-NT reporter construct containing the intergenic region between *hrpR* and *hrpH* including a deletion of AauR-binding site Rbm in the promoter of *hrpR* fused with a promoterless *gfp*	This study
$phrpL_{promoter}$-gfp	pPROBE-NT reporter construct containing the intergenic region between *hrpL* and *hrpJ* including the promoter of *hrpL* fused with a promoterless *gfp*	(Yan *et al.* 2019)
$pavrRpm1_{promoter}$-gfp	Reporter construct containing *avrRpm1* fused with a C-terminal hemagglutinin tag followed by a promoterless *gfp*, with both genes driven by the native *avrRpm1* promoter in vector pBBR1MCS-2.	(Chang et al. 2005)
$paatJ_{promoter}$-gfp	pPROBE-NT reporter construct containing the promoter of *aatJ* fused with a promoterless *gfp.*	This study
pME6010	pACYC177-pVS1 *E. coli* and *Pseudomonas* spp. shuttle vector, Tc^r	(Heeb *et al.* 2000)
pME6010-aauS	pME6010 containing an intact *aauS* gene, used to restore the mutation of *aauS* in DC3000. Tc^r	This study
pME6010-aauR	pME6010 containing an intact *aauR* gene, used to restore the mutation of *aauR* in DC3000. Tc^r	This study
pME6010-aatJ	pME6010 containing an intact *aatJ* gene, used to restore the mutation of *aatJ* in DC3000. Tc^r	This study
pET28a:aauR	Expression of recombinant AauR in *E. coli*	This study

[#]: Rif^r: rifampicin; Tc^r, tetracycline resistance; Km^r, kanamycin resistance; Cm^r, chloramphenicol resistance
