# Supplementary material for: Ancient co-option of an amino acid ABC transporter locus in Pseudomonas syringae for host signal-dependent virulence gene regulation
Source: PLoS Pathog. 2020 Jul 16;16(7):e1008680. doi: 10.1371/journal.ppat.1008680 (PMC7386598; doi:10.1371/journal.ppat.1008680)
Supplement: S2 Table — (PDF) [file ppat.1008680.s017.pdf]

S2 Table. Sequences of oligonucleotides used in this study.

**Oligonucleotides**	**DNA sequences (5’ to 3’)***
aauS-F1	atagaaTTCCGCACTTGAGCATCGTCG
aauS_OP_R1	GAA CAT GGC GTC CAG TGC ATT GCG CAT TTC ATC ACA GTC GTC C
aauS_OP_F1	GGACGACTGTGATGAAATGCGCAATGCACTGGACGCCATG TTC
aauS_R1	tataagCTTGAGACGGCTCAGCAGCTC
aauR_F1	atagaattCAATGCACTGGACGCCATGTTC
aauR_OP_R1	GAC CTT GTC AAA CAG TGT GGT CCT TGA GAC GGC TCA GCA GCT C
aauR_OP_F1	GAGCTGCTGAGCCGTCTCAAGGACCACACTGTTTGACAAG GTC
aauR_R1	tataagCTTCCAGTCACCAGACCCTGG
aauSR_OP_F1	GGACGACTGTGATGAAATGCGGACCACACTGTTTGACAAG GTC
aauSR_OP_R1	GAC CTT GTC AAA CAG TGT GGT CCG CAT TTC ATC ACA GTC GTC C
aatJ-F1	atagaatTCAGCAGCTATATGGACACCG
aatJ_OP_R1	GGTTGAACCGCTTACTCAGGCTGGCCGAGGGTGATAGTGC
aatJ_OP_F1	GCACTATCACCCTCGGCCAGCCTGAGTAAGCGGTTCAACC
aatJ-R1	tagaagCTTGTTCGCAGACCCGTGCAG
aatP_F1	atagaaTTCGCTCGATCCCGCTGCTG
aatP_OP_R1	GCTTGCTTAGTGCTGGAGAATCACTTGTTGACGTTCTTGAT GG
aatP_OP_F1	CCATCAAGAACGTCAACAAGTGATTCTCCAGCACTAAGCA AGC
aatP_R1	tagaagCTTCACCCTGATAACTGTCGGC
aatQM_F1	tagaaGCTTTCATGATGGACGACGC
aatQM_OVLP_R1	GCAAAGCTGATCGTGAAATACCGGTGGACTTGAAGAACAC G
aatQM_OVLP_F1	CGTGTTCTTCAAGTCCACCGGTATTTCACGATCAGCTTTGC
aatQM_R1	atagaaTTCGTCGAACAGCATGACCAC
B728a_aauR_F3_OVLPf_18	cctgcaggtcgactctagagAACTGAGCAAGGCTGTCGATG
B728a_aauR_R3_OVLP	CGCAGTGCTCAAAGCCCGTAAGGTCGCTGTTTATGCTCATG G
B728a_aauR_F4_OVLP	CCATGAGCATAAACAGCGACCTTACGGGCTTTGAGCACTG CG

B728a_aauR_R4_OVLPr_18	tgattacgaattcgagctcgTTCAGCGACCTGAAAGACCC
hrpR-F1_pro	gcctgcaggtcgactctagaGCTTACAAACCGGATACAGTTGCC
hrpR-R1_pro	gaataagccgaattcgagctcgCTCTCATGGTGGGTGGCAAGC
hrpR-F1	tataaGCTTACAAACCGGATACAGTTGCC
hrpR-R1	ataggtacCTCTCATGGTGGGTGGCAAGC
hrpR_F6_OVLP	GTCTAAGCAGGTGGGCTTTTGTGCGATGATGACTAAATGCC
hrpR_R7_OVLP	GGCATTTAGTCATCATCGCACAAAAGCCCACCTGCTTAGAC
hrpR_F6_OVLP	GTCTAAGCAGGTGGGCTTTTGTGCGATGATGACTAAATGCC
hrpR_R6	tagaagctTCAAAGCAGCTGTGATCCG
hrpR_F7	atagaattcGTTTTTGCCAGTGATCCACG
hrpR_R7_OVLP	GGCATTTAGTCATCATCGCACAAAAGCCCACCTGCTTAGAC
aatJ_F2	TCAAAGACATCCTCTGGCGC
aatJ_R2	atagaattCAGATGGGGAACGATACGC
hrpR-F1_pro	gcctgcaggtcgactctagaGCTTACAAACCGGATACAGTTGCC
pME6010-F1	TGGTACCCGGGAGCTCGAATTC
pME6010-R1	GCATCGATAGATCTCGAGCAAGACG
pME6010-F2	GATCCAACCCCTCCGCTGCTATAG
pME6010-R2	CTATAGCAGCGGAGGGGTTGGATC
#4176-F	CTCGAGATCTATCGATGCCGACATGAGAAGAAAGCC
#4176-R	TCGAGCTCCCGGGTACCATCAAAGCCCATATTTCTTG
#4171-F	ACGGGAAACGTCTTGCCCTCTCATTGCGGCTCTATC
#4171-R	CATGCATCGATAGATCATCAGTTCAGGGTGGTTGAAC
#4175-F	ACGGGAAACGTCTTGCACCCTCTCAGGCAGCTGTTC
#4175-R	CATGCATCGATAGATCAGTTCGCTGTTGATGCTCATG
*pET28a*_F	CATGGTATATCTCCTTCTTAAAG
*aauR_28a*_F	CTTTAAGAAGGAGATATACCATGAGCATCAACAGCGAACTC
*aauR_28a*_R	GTGCTCGAGTGCGGCCGCAAGAAGCCCATATTTCTTGACCTTG
*attJ*_F	CCTGAATATTCGACTGCATTCGGCTTTCCGAACGCGTCTCCCTGCGCGAT
*attJ*_R	ATCGCGCAGGGAGACGCGTTCGGAAAGCCGAATGCAGTCGAATATTCAGG

*attJΔRbm*_F	TCCCTCTCCTGAATATTCGACTGCACGCGTCTCCCTGCGCGATTCGGTTT
*attJΔRbm*_R	AAACCGAATCGCGCAGGGAGACGCGTGCAGTCGAATATTCAGGAGAGGGA
*hrpRS*_F	TAAGCAGGTGGGCTTTTGTTCGGATTGCCGAATGCGATGATGACTAAATG
*hrpRS*_R	CATTTAGTCATCATCGCATTCGGCAATCCGAACAAAAGCCCACCTGCTTA
*hrpRSΔRbm*_F	AATAGTCTAAGCAGGTGGGCTTTTGTGCGATGATGACTAAATGCCAGTAC
*hrpRSΔRbm*_R	GTACTGGCATTTAGTCATCATCGCACAAAAGCCCACCTGCTTAGACTATT

*: Restriction sites used for cloning are underlined in oligonucleotides.
