# Supplementary material for: Ancient co-option of an amino acid ABC transporter locus in Pseudomonas syringae for host signal-dependent virulence gene regulation
Source: PLoS Pathog. 2020 Jul 16;16(7):e1008680. doi: 10.1371/journal.ppat.1008680 (PMC7386598; doi:10.1371/journal.ppat.1008680)
Supplement: S3 Table — (PDF) [file ppat.1008680.s018.pdf]

S3 Table. Metadata of *P. syringae* genomes and individual gene sequences used for phylogenetics

Full_Name	Species_Name	Isolate/strain	Assembly_Status	Assembly_Accessi	AatJ_Locus	AauR_Locus	HrpR_Locus	Phylogroup	Ref_for_Alignment?	Canonical T3SS?
P_aeruginosa_PAO1	Pseudomonas aeruginosa	PAO1	Complete Genome	GCA_000006765.1	PA1342	PA1335	NA	0	N	N
P_fluorescens_Pf0-1	Pseudomonas fluorescens	Pf0-1	Complete Genome	GCA_000012445.1	Pfl01_4535	Pfl01_4540	NA	0	N	N
P_fluorescens_SBW25	Pseudomonas fluorescens	SBW25	Complete Genome	GCA_000009225.1	PFLU_1139	PFLU_1134	NA	0	N	N
P_protegens_Pf-5	Pseudomonas protegens	Pf-5	Complete Genome	GCA_000012265.1	PFL_4871	PFL_4876	NA	0	N	N
P_putida_KT2440	Pseudomonas putida	KT2440	Complete Genome	GCA_000007565.2	PP_1071	PP_1066	NA	0	N	N
P_syringae_inb918	Pseudomonas syringae	inb918	Complete Genome	GCA_004006335.1	CT157_23070	CT157_23095	NA	0	N	N
P_cichorii_ICMP_1649	Pseudomonas cichorii	ICMP 1649	Scaffold	GCF_003699995.1	NA	ALQ47_RS04710	NA	11	N	N
P_cichorii_JBC1	Pseudomonas cichorii	JBC1	Complete Genome	GCA_000517305.1	PCH70_40000	PCH70_40050	NA	11	N	N
P_syringae_GAW0119	Pseudomonas syringae	GAW0119	Contig	GCF_000737245.1	IV01_RS16770	IV01_RS16745	NA	12	N	N
P_savastanoi_ICMP_2236	Pseudomonas savastanoi	ICMP 2236	Scaffold	GCF_003701285.1	ALP94_RS02575	ALP94_RS02550	NA	13	N	N
P_syringae_CC1557	Pseudomonas syringae	CC1557	Complete Genome	GCA_000452705.3	N018_05975	N018_05950	Un-annotated in GenBank	10b	N	Y
P_syringae_UB0390	Pseudomonas syringae	UB0390	Contig	GCF_000737225.1	IV03_RS06245	IV03_RS06270	NA	2c	N	N
P_viridiflava_CFBP_1590	Pseudomonas viridiflava	CFBP 1590	Complete Genome	GCA_900184295.1	CFBP1590__1386	CFBP1590__1381	NA	7a	N	N
P_viridiflava_ICMP_2848	Pseudomonas viridiflava	ICMP 2848	Scaffold	GCF_001642795.1	AO065_RS18900	AO065_RS18875	NA	7a	N	N
P_viridiflava_TA043	Pseudomonas viridiflava	TA043	Scaffold	GCF_000452485.1	N030_RS0127095	N030_RS0127120	NA	7a	N	N
P_viridiflava_ICMP_8820	Pseudomonas viridiflava	ICMP 8820	Contig	GCF_002723575.1	AO275_RS00865	AO275_RS00890	NA	7b	N	N
P_syringae_ATCC_10859	Pseudomonas syringae	ATCC 10859	Complete Genome	GCA_001482725.1	ACA40_19380	ACA40_19405	ACA40_06030	2	N	Y
P_syringae_CFBP3840	Pseudomonas syringae	CFBP3840	Complete Genome	GCA_900235815.1	CFBP3840_01258	CFBP3840_01253	CFBP3840_04094	3	N	Y
P_syringae_CFBP6109	Pseudomonas syringae	CFBP6109	Complete Genome	GCA_900235885.1	CFBP6109_00894	CFBP6109_00888	CFBP6109_04871	3	N	Y
P_amygdali_M301315	Pseudomonas amygdali	M301315	Complete Genome	GCA_000146005.2	PLA107_006305	PLA107_006280	PLA107_018915	3	N	Y
P_savastanoi_1448A_BAA-978	Pseudomonas savastanoi	1448A; BAA-9	Complete Genome	GCA_000012205.1	PSPPH_3902	PSPPH_3907	PSPPH_1270	3	Y	Y
P_coronafaciens_ICMP8921	Pseudomonas coronafaciens	ICMP8921	Scaffold	GCF_001401235.1	ALO38_RS25180	ALO38_RS25155	ALO38_RS01990	4	N	Y
P_coronafaciens_ICMP9088	Pseudomonas coronafaciens	ICMP9088	Scaffold	GCF_001400895.1	ALO57_RS12770	ALO57_RS12795	ALO57_RS16785	4	Y	Y
P_coronafaciens_ICMP4457	Pseudomonas coronafaciens	ICMP4457	Scaffold	GCF_001400695.1	ALO66_RS08985	ALO66_RS08960	ALO66_RS13930	4	N	Y
P_coronafaciens_ICMP4323	Pseudomonas coronafaciens	ICMP4323	Scaffold	GCF_001400345.1	ALO77_RS05930	ALO77_RS05905	ALO77_RS08370	4	N	Y
P_syringae_CC1513	Pseudomonas syringae	CC1513	Contig	GCF_000452765.1	N014_RS0208970	N014_RS0208945	N014_RS0216560	4	N	Y
P_syringae_YM7930	Pseudomonas syringae group genomosp. 3	YM7930	Scaffold	GCF_001293575.1	AC507_RS10550	AC507_RS10575	AC507_RS03155	5	N	Y
P_cannabina_ICMP2823	Pseudomonas cannabina	ICMP2823	Scaffold	GCF_001400175.1	ALO81_RS24130	ALO81_RS24105	ALO81_RS06615	5	N	Y
P_syringae_ICMP15200	Pseudomonas syringae	ICMP15200	Scaffold	GCF_001401375.1	ALO83_RS11875	ALO83_RS11850	ALO83_RS14375	5	N	Y
P_syringae_ES4326	Pseudomonas syringae group genomosp. 3	ES4326	Scaffold	GCF_000145845.1	PMA4326_RS05030	NA	PMA4326_RS03600	5	N	Y
P_syringae_ICMP4531	Pseudomonas syringae group genomosp. 7	ICMP4531	Scaffold	GCF_001400835.1	ALO68_RS19110	ALO68_RS27035	ALO68_RS10975	6	N	Y
P_caricapapayae_ICMP2855	Pseudomonas caricapapayae	ICMP2855	Scaffold	GCF_001400735.1	ALO80_RS14230	ALO80_RS03565	ALO80_RS24090	6	N	Y
P_viridiflava_ICMP_19473	Pseudomonas viridiflava	ICMP 19473	Scaffold	GCF_003702045.1	ALP40_RS10635	ALP40_RS10660	ALP40_RS13960	8	N	Y
P_syringae_CC1583	Pseudomonas syringae	CC1583	Contig	GCF_000452665.1	N020_RS0204970	N020_RS0204945	N020_RS02000000226055	10a	Y	Y
P_syringae_CC1466	Pseudomonas syringae	CC1466	Contig	GCF_000452785.1	N013_RS0201020	N013_RS0200995	N013_RS02000000226405	10b	Y	Y
P_syringae_DC3000	Pseudomonas syringae group genomosp. 3	DC3000	Complete Genome	GCA_000007805.1	PSPTO_4171	PSPTO_4176	PSPTO_1379	1a	Y	Y
P_syringae_ICMP_9617	Pseudomonas syringae	ICMP 9617	Chromosome	GCF_000658965.1	A250_RS19390	A250_RS19420	A250_RS06240	1b	Y	Y
P_syringae_NZ-45	Pseudomonas syringae	NZ-45	Complete Genome	GCA_001913215.1	PsaNZ45_21750	PsaNZ45_21780	PsaNZ45_06775	1b	N	Y
P_syringae_CFBP6411	Pseudomonas syringae group genomosp. 3	CFBP6411	Complete Genome	GCA_900235905.1	CFBP6411_04297	CFBP6411_04302	CFBP6411_01334	1c	Y	Y
P_syringae_31R1	Pseudomonas syringae	31R1	Chromosome	GCF_900105295.1	BLV36_RS14660	BLV36_RS14685	BLV36_RS00695	2a	Y	Y
P_syringae_Cit_7	Pseudomonas syringae	Cit 7	Scaffold	GCA_000145825.1	PSYCIT7_01120	PSYCIT7_32071	PSYCIT7_05430	2a	N	Y
P_syringae_UMAF0158	Pseudomonas syringae	UMAF0158	Complete Genome	GCA_001281365.1	PSYRMG_10370	PSYRMG_10395	PSYRMG_22330	2a	N	Y
P_syringae_PP1	Pseudomonas syringae	PP1	Complete Genome	GCA_000452445.3	N032_20590	N032_20615	N032_07030	2b	N	Y
P_syringae_SM	Pseudomonas syringae	SM	Chromosome	GCF_000412165.1	PSSSM_RS06385	PSSSM_RS06360	PSSSM_RS19580	2b	Y	Y
P_syringae_Pss9097	Pseudomonas syringae	Pss9097	Complete Genome	GCA_002905815.2	BKC06_020255	BKC06_020280	BKC06_006480	2d	N	Y
P_syringae_B728a	Pseudomonas syringae	B728a	Complete Genome	GCA_000012245.1	Psyr_3908	Psyr_3913	Psyr_1190	2d	Y	Y
P_syringae_B301D	Pseudomonas syringae	B301D	Complete Genome	GCA_000988485.1	PsyrB_20290	PsyrB_20315	PsyrB_06270	2d	N	Y
P_syringae_BS2730	Pseudomonas syringae	BS2730	Scaffold	GCF_004362325.1	BCD77_RS04560	BCD77_RS04535	BCD77_RS01810	5b	N	Y
P_syringae_0788_9	Pseudomonas syringae	0788_9	Scaffold	GCF_001293775.1	ABJ99_RS07570	ABJ99_RS07545	ABJ99_RS21355	5c	N	Y
P_syringae_ICMP3956	Pseudomonas syringae group genomosp. 3	ICMP3956	Scaffold	GCF_001400635.1	ALO52_RS13035	ALO52_RS13060	ALO52_RS09725	7a	N	Y
P_viridiflava_CFBP13507	Pseudomonas viridiflava	CFBP13507	Contig	GCF_005233675.1	PviCFBP13507_RS07705	PviCFBP13507_RS07680	PviCFBP13507_RS04245	7a	N	Y
P_syringae_CC1417	Pseudomonas syringae	CC1417	Contig	GCF_000452825.1	N011_RS0209770	N011_RS0209745	N011_RS0203430	9a	N	Y
P_syringae_CC1524	Pseudomonas syringae	CC1524	Contig	GCF_000452745.1	N015_RS0212175	N015_RS0212150	N015_RS0216545	9a	N	Y
P_asturiensis_LMG_26898	Pseudomonas asturiensis	LMG 26898	Scaffold	GCF_900143095.1	BUB69_RS20990	BUB69_RS20965	BUB69_RS06515	9b	Y	Y
