# Supplementary material for: Ancient co-option of an amino acid ABC transporter locus in Pseudomonas syringae for host signal-dependent virulence gene regulation
Source: PLoS Pathog. 2020 Jul 16;16(7):e1008680. doi: 10.1371/journal.ppat.1008680 (PMC7386598; doi:10.1371/journal.ppat.1008680)
Supplement: Attachment — Submitted filename: PPATHOGENS-D-20-00481_Author_Response.pdf [file ppat.1008680.s020.pdf]

Oregon State University

Botany and Plant Pathology
Oregon State University
2082 Cordley Hall
Corvallis, Oregon 97331

P 541-737-3451
F 541-737-3573
bpp.oregonstate.edu

6/1/2020

Dear Dr. Mackey,

We thank you and the reviewers for your insightful comments and suggestions to improve our manuscript PPATHOGENS-D-20-00481 "Ancient co-option of an amino acid ABC transporter locus in *Pseudomonas syringae* for host signal-dependent virulence gene regulation."

We are now submitting a revised manuscript that includes edits requested by you and the reviewers. We also removed three references to 'data not shown' as requested by the PLOS editorial staff. All changes to our manuscript were tracked and can be seen in the uploaded tracked edit version.

Below are our detailed responses to each question and comment.

Again, thank you for your assistance with our manuscript.

Sincerely,

Jeff Anderson
Oregon State University

1. More detail describing the screen would be useful, including indication of other genes identified.

We have included additional details of our Tn5 screen in the Methods section, including screening conditions used to identify mutants with Tn5 insertions in the *aat/aau* locus, which will allow readers to understand the basis of our genetic screen and replicate our findings.

From this screen we identified mutants with insertions in all known positive regulators of T3SS, including *hrpR*, *hrpS*, *rpoN*, and *rhpS*, thus validating that our screening method was robust. We are planning a separate manuscript that describes our methods for shotgun sequencing of Tn5 libraries, as well as a bar-coding approach to assigning specific Tn5 insertions to specific mutant strains. This manuscript would include a full list of Tn5 insertion sites identified to demonstrate the effectiveness of this approach. For this reason, and because we did not use any Tn5 mutant strains for experiments in this work, we would prefer to keep the focus of this manuscript on *aat*/*aau* rather than the overall results of the genetic screen.

2. The key gene induction assays in figure 1 would be strengthened by addition of a genetic negative control (e.g. a hrpR/S mutant) in addition to the sucrose only treatment.

We agree that including a genetic negative control would strengthen these results by demonstrating that *aat*/*aau* mutant phenotypes are indeed intermediate. Unfortunately, we do not have these data in hand and, given current lab work restrictions due to pandemic, the timeline for completing these experiments is uncertain.

3. Inclusion of a genetic negative control also would provide better context for the magnitude of growth defects in the key bacterial growth assay in figure 2C.

We agree that this control would be informative, however we do not have data directly comparing growth of *aat*/*aau* mutants with a T3SS-deficient mutant and, as explained above, the timeline for completing these experiments in the laboratory is uncertain. In separate experiments, we have tested growth of a *hrcC*- mutant, and generally see a 2-3 log decrease in growth compared to wild type 3 days post-inoculation (consistent with most reports in the literature). Based on the more modest 0.5-1.0 log decrease seen with *aat*/*aau* mutants, it is likely that virulence is only partially compromised.

4. Rather than representative, single experiments, data for bacterial growth should be a composite of multiple, independent experiments.

All *in planta* bacterial growth data are now pooled from multiple experiments.

For Figure 2C, bacterial growth measurements were done as six independent experiments. In two experiments, we included all three mutant strains (*aauR*-, *aauS*- and *aatJ*-) and their

respective complemented strains. In this revised manuscript, data shown in Fig. 2C are now pooled from these two experiments. In the remaining four experiments for Fig 2C, we tested either *aauS*- and *aauR*- mutants (two experiments), or the *aatJ*- mutant (two experiments), along with respective complemented strains. Because we did not test all three mutants in these four experiments, in our opinion it would be inappropriate to pool these data with those shown in Fig. 2C. Therefore, we have elected to include data from the four smaller experiments as Fig. S3B.

5. Phospho-point mutants requested by reviewer #2 are beyond the scope expected for this study.

6. Presenting in vitro bacterial growth as the OD600 without log transformation is OK.

Part I - Summary
Please use this section to discuss strengths/weaknesses of study, novelty/significance, general execution and scholarship.

Reviewer #1: The manuscript by Yan et al. identifies a two-component system and substrate binding protein that contributes to the regulation of the type III secretion system in Pseudomonas syringae. The system is conserved across P. syringae species and regulates the type III secretion system in response to host metabolites (amino acids aspartate and glutamate). Overall the manuscript is thorough and well written. I only have minor comments.

Reviewer #2: The authors have identified a two component system and associated SBP that drive full expression of hrp regulon and P syringae virulence in response to acidic amino acids through direct regulation of hrpR transcription. The aauS sensor kinase, aauR response regulator, and aatJ SBP all play similar non-additive roles in contributing to hrp regulon expression in response to asp and glu but hrp regulation does not require the ABC transporter genes for asp/glu in vitro or in vivo. The authors find a consensus aauR box (Rbm) upstream of hrpR and demonstrate that aauR binding to the hrpR promoter is dependent on the Rbm. Rbm mutants phenocopy an aauR mutant indicating that the aauR hrp regulation phenotypes can be adequately explained by the hrpR Rbm site. The hrpR Rbm site is (almost) perfectly conserved among P. syringae in all phylogroups with a canonical T3SS. Similar mutant phenotypes were observed with both Pto and Pss. The appropriateness of glu/asp as an accurate signal of the plant host environment and the potential evolutionary context of AauR/S-HrpR/S regulation is discussed.

Reviewer #3: This study reveals a novel regulatory mechanism controlling the expression of the Type III secretion system in the bacterial pathogen Pseudomonas syringae. The study is thorough and complete and makes a significant new contribution to our understanding of the complex mechanisms by which plant pathogens sense and respond to their environment to regulate expression of virulence factors. The amount of data presented is substantial, the experiments are carefully done and well-controlled, and the manuscript is clearly written. Overall, the claims are well-supported by the data, and as

described below, I have only a few scientific and editorial comments that the authors should consider when revising their manuscript.

Part II – Major Issues: Key Experiments Required for Acceptance

Reviewer #1: (No Response)

Reviewer #2: This is one of those excellent papers where, even with a critical eye, I find very little to fault with this work. The genetics are solid with appropriate complementation and controls. The experiments are carefully and thoroughly designed. Multiple forms of evidence are used to support major points both in vivo and in vitro.

To complement the Rbm analysis I would like to see an analysis of genetic variation of the aatJ-aauR locus within the same P. syringae as well as in other pseudomonads.

We now include phylogenetic analyses of *aatJ* and AauS as supplemental figures 12 and 13. Similar to the *hrpR* phylogeny, the *aatJ* and AauS trees show vertical inheritance. We have added text describing these data in Results section line 269. We note that previous studies showed that the *aat*/*aau* locus is conserved across diverse pseudomonads – we now reference this directly on line 269.

Other than that, I have only minor concerns.

Reviewer #3: 1. Fig. 2A. How is expression of AvrPto in planta normalized? Were bacterial numbers in the leaf tissue analyzed from the same samples?

For Fig 2A we normalized gel loading based on total protein in extracts from infected tissue (using CBB staining to confirm). Yes, we measured bacterial numbers from these same samples, and we now include these data as Fig. S3A. No difference in bacterial numbers was observed at this early time point.

Part III – Minor Issues: Editorial and Data Presentation Modifications

Reviewer #1: Regarding the screen
-was this screen successful in finding components that are already known to play a role in general T3SS/T3E expression?

Yes, we identified Tn5 insertions in coding sequences or promoter regions of essentially all known positive regulators including *hrpR*, *hrpS*, *rpoN* (promoter insertion), *hrpL*, and *rhpS*.

-how was reduced GFP fluorescence scored/what was the cutoff?

For 1st round of screening, we visually scored 20,000 individual colonies for loss of GFP fluorescence using a stereomicroscope. We picked any colonies that by eye showed decreased fluorescence. From this 1st round, we selected 400 mutants and did a 2nd round of screening using 96- and 384-well liquid culture assays, with a cut-off of >20% decrease in GFP fluorescence relative to a wild type control strain. From this 2nd round, 200 mutants were selected, and gDNA pooled for sequencing to identify Tn5 insertion sites.

-would intermediate levels of GFP fluorescence correspond to mutations that play intermediate roles in T3SS expression?

Yes, we presume this to be the case, although we are careful not to draw conclusions from individual Tn5 mutants, as the position of transposon insertion may have varying effects on gene expression.

Figures

Figure 2C- how many leaves per plant for growth assays?

Each sample is bacteria isolated from 3 leaf disks cut from 3 leaves of a single plant. Rather than add this detail redundantly to multiple figure legends, we have included this information within revised Methods.

Figure 2D- based on the symptoms in the one picture shown, we might expect a larger difference in bacterial growth (completely green vs. chlorotic). Are these representative? Same for Figure 5F and G.

Yes, images are representative of disease symptoms observed in multiple independent experiments. We note that in our growth chamber, with environmental conditions typical for pathogen assays (22 C, 10 hour days), DC3000 grows to a maximum of ~1-5 x 10^7 cfu/cm^2 by day 3, from a starting population between 1 x 10^3 and 1 x 10^4 cfu/cm^2. In our experience, growth and disease symptoms are not linearly correlated -- under our assay conditions the threshold population necessary for disease symptom development with DC3000 is ~1 x 10^7 cfu/cm^2. The ~0.5-1.0 log decrease in growth for *aat*/*aau* mutants is sufficient to largely eliminate symptom development.

Figure 3- How close are the amino acid concentrations used in this study to physiological concentrations (aspartate, glutamate, serine, glutamine)?

Estimates of amino acid concentrations within the apoplast vary between studies and plant species, but generally are in the low micromolar range (10-500 uM). From one study of tomato, aspartate and glutamate were both present in apoplast at 150 uM, whereas serine and glutamine were ~ 50 uM [Rico and Preston (2008) *Mol Plant-Microbe Interact* 21:269–282].

Figure S5; lines 191-193, "Growth of all three mutant strains could be restored by introduction of plasmids carrying respective wild type alleles of each mutated gene (S5 Fig)." aauR complementation doesn't seem to show this exactly, growth rate starts the same, but then diverges from DC3000? Explain.

We do not know the molecular basis for partial complementation of *aauR* at later timepoints. Increased copy number of plasmid-borne *aauR* could lead to over-accumulation of AauR protein relative to WT cells, which in turn could impact growth either through potential feedback regulation or pleotropic effects on growth. We note that it is not uncommon for plasmid-based expression to only partially complement phenotypes of mutant strains (Rahme et al (1997) *PNAS* 94:13245; Mandin et al. (2005) *Mol Microbiol* 57:1367).

Overall it would have been ideal to have mutant in a known type III secretion system regulator as a control for comparison since the aauS, aauR and aatJ mutants seem intermediate in phenotype (eg. HrpL mutant)

We agree that including a genetic negative control would strengthen these results by demonstrating that *aat*/*aau* mutant phenotypes are indeed intermediate. Unfortunately, we do not have these data in hand and, given current lab work restrictions due to the pandemic, the timeline for completing these experiments is uncertain.

Reviewer #2: Why were only 14 of the 20 L amino acids tested? Not a huge deal but it's an odd omission.

These are the amino acids that we had on hand at the time of these experiments. Our goal was to assess specificity of *aatJ* induction by asp/glu, and at the time of these experiments we felt that 13 different amino acids, in addition to L-asp and L-glu, would be sufficient to demonstrate specificity of response.

Likewise a good positive control could have been added to AlphaScreen protein-DNA interaction experiment. Engineered phospho-residue point mutations in aauS and aauR would have lent strong support for the model.

Thank you for this suggestion. We agree that the Alpha screen method will be useful for testing phosphor-activation of AauS and AauR.

Lastly, I would have preferred to see more explicit descriptions of hypotheses for the role of AatJ in the regulatory cascade.

We have modified text within the Discussion (starting at line 331) to more explicitly describe possible roles of AatJ in activating AauSR.

references 17 and 51 are duplicates

Thank you – corrected.

Reviewer #3: Minor Scientific comments
2. Figure 1. It is interesting that the reduction of expression of hrpR/Sprom-gfp in the aau mutants is less dramatic that for expression of avrRpm1 and hrpL. Do you have any idea as to why this is the case?

Yes, this is an interesting observation that suggests signal amplification occurs in the pathway, with relatively modest changes in *hrpRS* expression resulting in larger changes in *hrpL*/*avrRpm1* expression. We do not know the basis for this difference, but in the future it would be interesting to use inducible constructs to assess how varying levels of *hrpRS* expression affect downstream gene expression.

3. Line 168 and Fig S4.The conclusion that the decreased growth of the mutants in in leaf tissue is not due to a general fitness defect cannot be made based on the observation that no difference in growth of the mutants was detected in KB or LB. Growth of the mutants should also be carefully assessed in defined media that may more closely resembles conditions in the apoplastic space. I think the relevant growth information is presented in a later figure (fig 3D?). Perhaps that data could be presented or referred to earlier in the results section?

Line 169: We now refer to data in Fig. 3D to support this conclusion.

4. Fig 3c and others. Bacterial growth, which is exponential, should be plotted on a logarithmic scale. All of the in-culture growth curves are plotted on a linear scale. I don't believe this is appropriate.

We agree that graphing bacterial growth on log scale is valuable because it allows for calculation of exact growth rates, comparable between experiments. In this case, because we are comparing overall growth characteristics between strains within an experiment rather than growth rates, we would prefer to keep data graphed on an arithmetic scale. We note that data used for graphing are provided in supplemental S1 data, allowing readers to assess growth using preferred method.

5. Fig. 3C. The growth delay observed for the aauR, aauS and aatJ mutants in M9 +

glutamate is interesting, and suggests that the aat/aau locus is required in part for the normal uptake of extracellular amino acids. Why is there only a delay in growth, as opposed to a more severe inhibition of groth? Is an aat/aau-independent mechanism induced to compensate for the absence of aat/aau?

We do not know why growth of *aat*/*aau* mutants on glutamate is not completely abolished, but we speculate that *P. syringae* may have alternative transport mechanisms for glu/asp that function in the absence of transport through AatQMP. It is not uncommon bacteria to have multiple uptake mechanisms for acidic amino acids. For instance, *E. coli* has at least three uptake mechanisms for glutamate and/or aspartate [Singh and Rohm (2008) *Microbiology* 154:797].

6. Figs 5A and 6A: the AauR binding motif is located quite a bit upstream of the translation start site for hrpR. This seems like a very long distance away for a regulatory motif. Does this suggest that there is a long 5'UTR? Or a binding site at a distance from the promoter, with possible DNA looping? Is there any data in the public domain that might provide you information about the transcription start site for the hrpR/S operon (RNA seq read, perhaps)? This is not a major concern, but some discsussion about the long distance should be included, especially since it is strongly conserved amongst the P. syringae genomes analyzed here.

We agree that the distance between Rbm and cds of HrpR is unusually large, suggesting that either looping of DNA or a long 5' UTR. Using public RNA-seq datasets [Nobori et al (2018) PNAS 115:E3055 and Lovelace et al. (2018) MPMI 31:750], there are reads that align to this region of the *hrpR*S promoter, yet are only a fraction of the read depth (~10%) observed for *hrpR* cds region. We hesitate to draw conclusions from these data because low levels of intergenic reads could be due to gDNA contamination of RNA-seq libraries. Despite this caveat, it is interesting that more reads do align to this *hrpR*S promoter region in bacteria isolated from leaves compared to bacteria cultured in MM (yet intergenic reads still represent only a very small fraction of total *hrpRS* reads), suggesting an alternative transcription start site may produce a mixed population of *hrpRS* transcripts. Future qPCR and 5'-RACE experiments will be informative to further dissect *hrpRS* promoter structure.

7. The hypothesis that AauS-AauR regulation of hrpRS may predate P. syringae speciation is intriguing. Could it also predate the speciation of P. syringae from other plant pathogenic Pseudomonas species?

This is a very interesting question. *P. syringae* likely evolved from a *P. fluorescens*-like ancestor and, because not all *P. syringae* carry the tripartite pathogenicity island (T-PAI), current models suggest that the T-PAI was acquired post-speciation [Xin et al, (2018) *Nature Rev Microbiol*, 16:316]. To our knowledge it is not known from where *P. syringae* acquired its T-PAI. There are existing *P. fluorescens* that carry a T3SS, but with some variation in composition and structure of the T3SS-encoding locus relative to the canonical T-PAI [Mavrodi et al (2011) *J Bacteriol* 193(1):177-89]. We are currently investigating if

Rbm (or Rbm-like motifs) are present upstream of genes orthologous to *hrpR* in these strains.

Editorial comments

1. The manuscript is well-written, engaging and easy to read. However, I did not find that enough basic information regarding how the mutant screen was carried out is provided in the Results section. I had to piece this together from the methods, and from referring to Turner et al (Ref. 18). I realize that PLoS journals aim for shorter manuscripts, but I wish more info had been provided up front.

In the revised Methods we now provide additional details about the protocol used for our Tn5 mutagenesis screen.

2. I suggest using the terms Microbial associated molecular patterns (MAMPs) rather than PAMPs.

Line 68: We included MAMPs as an alternate acronym within the introduction.

3. Fig 2 figure legend. I suggest adding the qualifier “normal level of virulence” between “….required for” and “DC3000 virulence”. A similar change should be made for the legend for Fig. 5. The mutants exhibit only a partial reduction in virulence.

Fig. 2 legend title corrected to “maximal level of DC3000 virulence” and Fig. 5 legend title corrected to “maximal virulence of DC3000”.

4. Figure 5E. This graph is hard to interpret, as one cannot see any data point value other than the biotin-DNA in the hrpRS strain. Can the axis be altered to show the low values? Or “nd” for none-detected if that is the case? It is hard to know how many data points are actually presented in the graph.

We altered the scale of the Y-axis in Fig. 5E to show low values.

5. Line 390. Is OD600 = 5.0 correct? Also, why was the culture shaken for four weeks? This seems like a very long time to grow a bacterial culture.

Yes, OD_{600} = 5.0 is correct. The $aatJ_{promoter}$:*gfp* strain showed higher levels of expression (relative to empty vector control) immediately after transferring to minimal medium, mostly likely due to residual activation by amino acids in KB medium, combined with stability of GFP protein. We maintained this culture for four weeks until levels of GFP fluorescence were reduced to background (empty vector) levels, providing maximum range and sensitivity to assess amino acid-induced increases in *aatJ* expression.

6. Inclusion of a diagram to illustrate the model for how AauS/AauR-dependent mechanism regulates expression of T3SS, to accompany the las section of the discussion, would be a nice addition to the manuscript.

We now include a diagram as Fig S15 and reference this model within the Discussion section.

PLOS authors have the option to publish the peer review history of their article (what does this mean?). If published, this will include your full peer review and any attached files.

If you choose "no", your identity will remain anonymous but your review may still be made public.

Do you want your identity to be public for this peer review? For information about this choice, including consent withdrawal, please see our Privacy Policy.

Reviewer #1: No

Reviewer #2: No

Reviewer #3: Yes: Barbara Kunkel

Figure Files:

While revising your submission, please upload your figure files to the Preflight Analysis and Conversion Engine (PACE) digital diagnostic tool, https://pacev2.apexcovantage.com. PACE helps ensure that figures meet PLOS requirements. To use PACE, you must first register as a user. Then, login and navigate to the UPLOAD tab, where you will find detailed instructions on how to use the tool. If you encounter any issues or have any questions when using PACE, please email us at figures@plos.org.

Data Requirements:

Please note that, as a condition of publication, PLOS' data policy requires that you make available all data used to draw the conclusions outlined in your manuscript. Data must be deposited in an appropriate repository, included within the body of the manuscript, or uploaded as supporting information. This includes all numerical values that were used to generate graphs, histograms etc.. For an example see here: http://www.plosbiology.org/article/info%3Adoi%2F10.1371%2Fjournal.pbio.1001908#s5.

Reproducibility:

To enhance the reproducibility of your results, PLOS recommends that you deposit laboratory protocols in protocols.io, where a protocol can be assigned its own identifier (DOI) such that it can be cited independently in the future. For instructions see http://journals.plos.org/plospathogens/s/submission-guidelines#loc-materials-and-methods
